# Mutation and Copy Number Alterations Analysis of KIF23 in Glioma

**DOI:** 10.3389/fgene.2021.646929

**Published:** 2021-05-03

**Authors:** Zheng Zhao, Zheng Wang, Zhao-Shi Bao, Wei-Zhen Gao, Yuan-Da Zhang, Ci-Jie Ruan, Tao Lv, Yong Wang, Li-Hua Sun

**Affiliations:** ^1^Beijing Neurosurgical Institute, Capital Medical University, Beijing, China; ^2^Department of Neurosurgery, Beijing Tiantan Hospital, Capital Medical University, Beijing, China; ^3^Department of Neurosurgery, Renji Hospital, Shanghai Jiao Tong University School of Medicine, Shanghai, China

**Keywords:** glioma, KIF23, malignancy, mutation, copy number alterations

## Abstract

In glioma, kinesin family member 23 (KIF23) is up-regulated and plays a vital role in oncogenesis. However, the mechanism underlying KIF23 overexpression in malignant glioma remains to be elucidated. This study aims to find potential causes of KIF23 high expression at genome level. To clarify this issue, we obtained point mutation and copy number alterations (CNAs) of KIF23 in 319 gliomas using whole-exome sequencing. Only two glioma samples with missense mutations in KIF23 coding region were identified, while 7 patients were detected with amplification of KIF23. Additional analysis showed that KIF23 amplification was significantly associated with higher expression of KIF23. Gene ontology analysis indicated that higher copy number of KIF23 was associated TNF-α signaling pathway and mitotic cell circle checkpoint, which probably caused by subsequent upregulated expression of KIF23. Moreover, pan-cancer analysis showed that gaining of copy number was significantly associated with higher expression of KIF23, consolidating our findings in glioma. Thus, it was deduced that elevated KIF23 expression in glioma tended to be caused by DNA copy number amplification, instead of mutation.

## Introduction

Kinesin family member 23 (KIF23) is a nuclear protein and plays a key role in regulating cytokinesis (Nislow et al., 1992; Zhu et al., 2005; Liu and Erikson, 2007). It has been found to be dysregulated and act as an oncogene with prognostic value in various tumors (Kato et al., 2016b; Iltzsche et al., 2017; Li X. L. et al., 2019). Our previous study showed that KIF23 mRNA expression was positively correlated with glioma grade, and high KIF23 expression conferred poor survival in glioma, which was further validated by the TCGA, REMBRANDT, and GSE16011 database (Sun et al., 2016). These results indicated that dysregulated KIF23 may play an essential role in tumorigenesis and progression, but how KIF23 expression is upregulated in cancers remains unelucidated.

Previous study showed that in the tumors with up-regulated KIF23 expression, DNA mutation of KIF23 was detected in nearly half of tested human cancer types, and CNAs of KIF23 showed gain in 30% of tested tumors (Cerami et al., 2012). Besides, p.P916R mutation of KIF23 causes a rare hereditary form of dyserythropoietic anemia (CDA III) with predisposition to blood cancer (Liljeholm et al., 2013), and they further demonstrated that overexpression of KIF23 in non-small-cell lung cancer might be caused by CNAs (Vikberg et al., 2017). The above studies indicated that KIF23 gene expression can be modulated either by DNA mutation or by CNAs. However, KIF23 mutation and CNAs status in glioma is unclear. Given the idea that KIF23 is a novel prognostic biomarker with potential therapeutic implications in glioma, it is valuable to investigate the mutation and CNAs status of KIF23 in glioma.

In this study, we screened for KIF23 DNA mutation and CNAs in 319 gliomas with DNA and RNA sequencing data, and demonstrated that elevated KIF23 expression in glioma was probably caused by DNA copy number amplification. In terms of the important role of KIF23 in tumorigenesis and malignant aggressive progression of glioma, further understanding of its functional mechanism and pathway should be investigated.

## Materials and Methods

### RNA-Sequencing Data

Two independent RNA-seq datasets (mRNAseq_325 and mRNAseq_693) and paired clinical information were obtained from Chinese Glioma Genome Atlas (CGGA) database^1^ (Zhao et al., 2021). In the two datasets, only samples with definite WHO classification were included for survival and grade expression pattern analysis. Thus, 321 glioma samples (103 WHO grade II, 79 WHO grade III and 139 WHO grade IV) of CGGA mRNAseq_325 dataset, 692 glioma samples (188 WHO grade II, 255 WHO grade III, and 249 WHO grade IV) of CGGA mRNAseq_693 dataset were enrolled for subsequent analysis.

### Whole-Exome Sequencing (WES) Data

Genomic DNA from tumor and matched blood sample was extracted and confirmed for high integrity by 1% agarose gel electrophoresis. The DNA was subsequently fragmented, quality-controlled, and then pair-end libraries were prepared. For whole exome sequencing, Agilent SureSelect kit v6 was used for target capture. Sequencing was done on Illumina Hiseq platform using 150 bp pair-end sequencing strategy. In total, 319 whole-exome sequencing data of glioma samples were obtained, which were also available at CGGA Network.

DNA sequencing data were then mapped to the reference human genome (UCSC hg19) using Burrows-Wheeler Aligner (version 0.7.12-r1039, bwa mem) (Li and Durbin, 2009) with default parameters. Then, SAMtools (version 1.2) (Li et al., 2009) and Picard (version 2.0.1, Broad Institute)^2^ were used to sort the reads by coordinates and mark duplicates. Statistics such as sequencing depth and coverage were calculated based on the resultant BAM files. SAVI2 was used to identify somatic mutations (including single nucleotide variations and short insertion/deletions) as previously described (Wang et al., 2016; Hu et al., 2018). In this pipeline, SAMtools mpileup and bcftools were used to perform variant calling, then the preliminary variant list was filtered to remove positions with no sufficient sequencing depth, positions with only low-quality reads, and positions that are biased toward either strand. Somatic mutations were identified and evaluated by an Empirical Bayesian method. In particular, mutations with significantly higher allele frequency in tumors than that in normal control were selected. Additionally, CNVkit (version 0.9.4.dev0)(Talevich et al., 2016) was used to detect copy number changes from WES data.

### Gene Set Enrichment Analysis

Firstly, we obtain the KIF23 CNV status WES data from CGGA database^3^ and Cancer Hallmarks associated geneset from GSEA website^4^ version 7.2. By integrating the WES data and matched RNA-seq data, we calculated the fold change of gene expression for each gene between KIF23 with or without CNV. Next, we use R package GSVA (version 1.36.3) to do enrichment analysis in cancer hallmarks.

### Statistical Analysis

Statistical analysis was performed using SPSS Graduate Pack (version 16.0) and GraphPad Prism (version 5.0) statistical software. Descriptive statistics were shown as mean ± standard deviation. Student’s t test, one-way ANOVA test were used to test the significance of differences. Overall survival time (OS) was calculated from the date of histological diagnosis until death or the last follow-up. Kaplan-Meier survival analysis was used to estimate the survival distributions, and log-rank test was used to assess the statistical significance between stratified survivals groups. Patients with KIF23 expression lower than median level of KIF23 was defined as low expression, while patients with higher than the median value or equal to the median one was defined as high expression. A two-sided *p* value < 0.05 was considered statistically significant.

## Results

### KIF23 Expression Is Positively Correlated With Tumor Grade and Confers Poor Survival in Glioma

**FIGURE 1:**
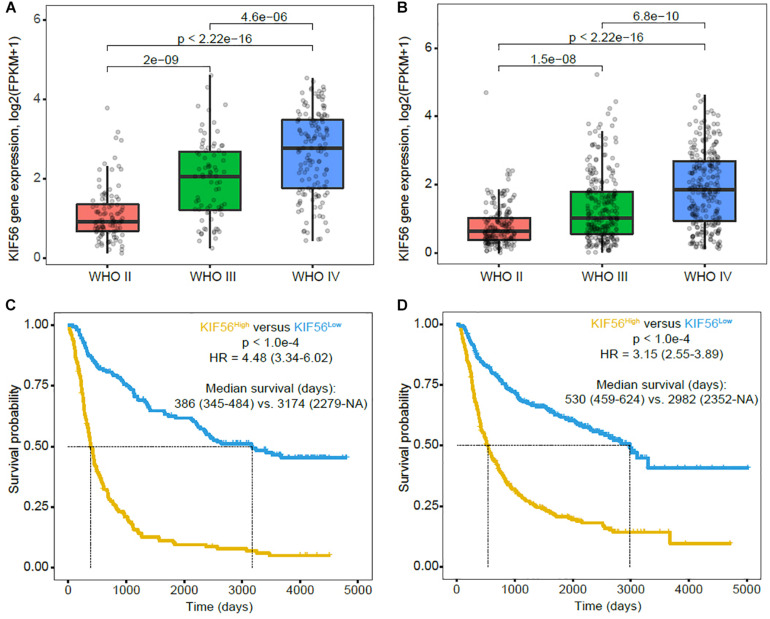
KIF23 expression pattern and prognostic value in CGGA RNA sequence database. **(A,B)** KIF23 expression is positively correlated with tumor grade. **(C,D)** High KIF23 confers a poor survival in glioma patients. High group, patients with higher KIF23 expression than the median value or equal to the median one. Low group, patients with lower KIF23 level than the median one.

To validate the results of our previous study (Sun et al., 2016), we analyzed KIF23 expression pattern using CGGA mRNAseq_325 and mRNAseq_693 datasets. We got the similar results that KIF23 expression was the highest in grade IV glioma group, while had the lowest expression in grade II glioma group (*p* < 0.001) (Figures 1A,B). Besides, we also found that patients with high KIF23 expression (median survival in CGGA_mRNAseq_325 dataset is 386 days, and CGGA mRNAseq_693 dataset is 530 days) had a significantly worse overall survival compared to those with low KIF23 expression (median survival in CGGA mRNAseq_693 dataset is 3174 days, and CGGA mRNAseq_693 dataset is 2982 days) (*p* < 0.001) (Figures 1C,D).

### Mutation Analysis of KIF23 in Glioma

**FIGURE 2:**
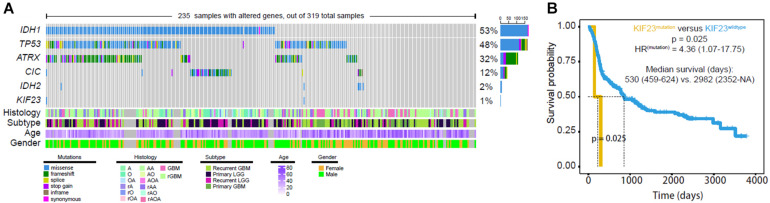
Mutation analysis was done in 319 glioma samples. Only two case-specific missense mutations were detected were detected **(A)** although mutation of KIF23 showed worse overall survival **(B)**.

Tumor DNA from 319 glioma patients were available for calling mutations, as shown in Figure 2. The mutation rate of IDH1 and TP53 was 53% and 48%, respectively. However, KIF23 mutation rate was less than 1% (Figure 2A). Only two case-specific missense mutations were detected, including non-synonymous change 69733357 G > A in Case104 and 69728081 C > T in Case241 (Table 1). Our results showed that patients with KIF23 mutation (median survival is 530 days) had a significantly worse overall survival compared to those without KIF23 mutation (median survival is 2982 days) (*p* = 0.025) (Figure 2B).

**TABLE 1:** Case-specific non-synonymous mutation of KIF23 in 2 glioma samples.

**Sample ID**	**Cancer type**	**Chromosome**	**Mutation position**	**Reference allele**	**Variant allele**	**AA change**
Case104	Recurrent GBM	15	69733357	G	A	G773D
Case 241	Recurrent GBM	15	69728081	C	T	P415S

### CNAs Analysis of KIF23 in Glioma

**FIGURE 3:**
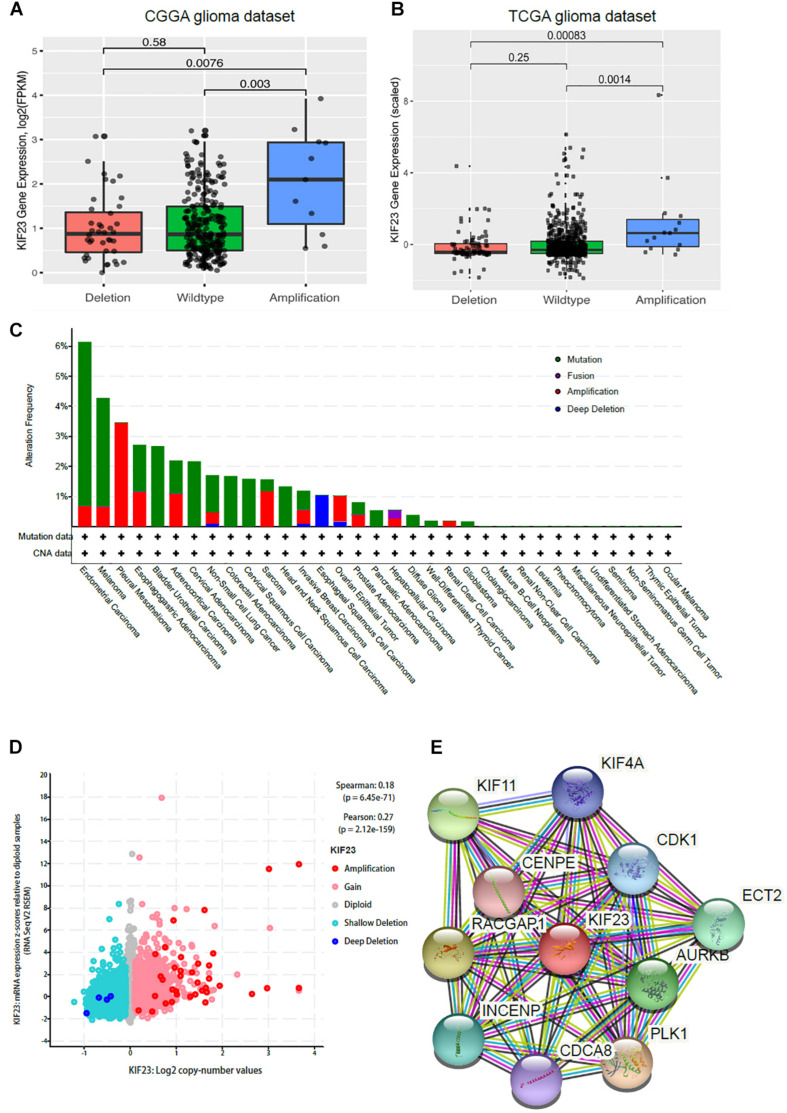
CNAs analysis was done in 319 glioma samples. KIF23 expression was highest in copy number amplification group in CGGA dataset **(A)** and further validated in TCGA dataset **(B)**. Copy number alteration as universally occurred in various cancers **(C)** and gaining of copy number was significantly associated with higher expression of KIF23 **(D)**. Network analysis revealed that KIF23 was tightly involved in mitosis **(E)**.

CNAs were analyzed from the above WES data with 319 samples. We defined log ratio > 0.25 as Amplification group (*n* = 11), log ratio < -0.25 as Deletion group (*n* = 40), others as Wildtype group (*n* = 268). As shown in Figure 3A, KIF23 expression was the highest in Amplification group. Though there’s no significant difference between Deletion group and Wildtype group, KIF23 in Wildtype group still showed relatively higher expression than that in Deletion group. The expression pattern was also independently validated in TCGA dataset (Figure 3B). Furthermore, we explored other focal CNAs in both low- and high-expression of KIF23 groups. We identified several significantly well-characterized genetic alterations in the case with high KIF23 expression (Table 2), such as PTEN loss (*p* value = 7.37e-8), CDKN2A loss (*p* value = 4.04e-12), CDKN2B loss (*p* value = 9.44e-12), KIT gain (*p* value = 3.33e-7), PDGFRA gain (*p* value = 5.06e-7), MET gain (*p* value = 1.30-04) and CDK6 gain (*p* value = 3.06e-04). Furthermore, we explore the KIF23 CNVs in Pan-cancer levels from TCGA datasets. Our results showed that KIF23 CNVs occurred in various human cancers (Figure 3C), including endometrial carcinoma, melanoma and gliomas, suggesting KIF23 as a critical role in pan-cancer. Moreover, pan-cancer analysis showed that gaining of copy number was significantly associated with higher expression of KIF23, consolidating our findings in glioma (Figure 3D). Network analysis revealed that KIF23 was tightly involved in mitosis, by interacting actively with genes such as CDK1 and CDCA8 (Figure 3E).

**TABLE 2:** Significant CNA events between KIF67 high expression and low expression groups.

**Alteration**	**Alt in exp^high^**	**Alt in exp^low^**	**WT in exp^high^**	**WT in exp^low^**	***P* value**
PTEN_loss	90	42	70	117	7.37e-08
CDKN2A_loss	104	42	56	117	4.04e-12
CDKN2B_loss	104	43	56	116	9.44e-12
KIT_gain	46	11	114	148	3.33e-07
PDGFRA_gain	47	12	113	147	5.06e-07
MET_gain	58	27	102	132	1.30e-04
CDK6_gain	51	23	109	136	3.06e-04

### Amplification of KIF23 Is a Negative Prognosticator for Glioma Patients

**FIGURE 4:**
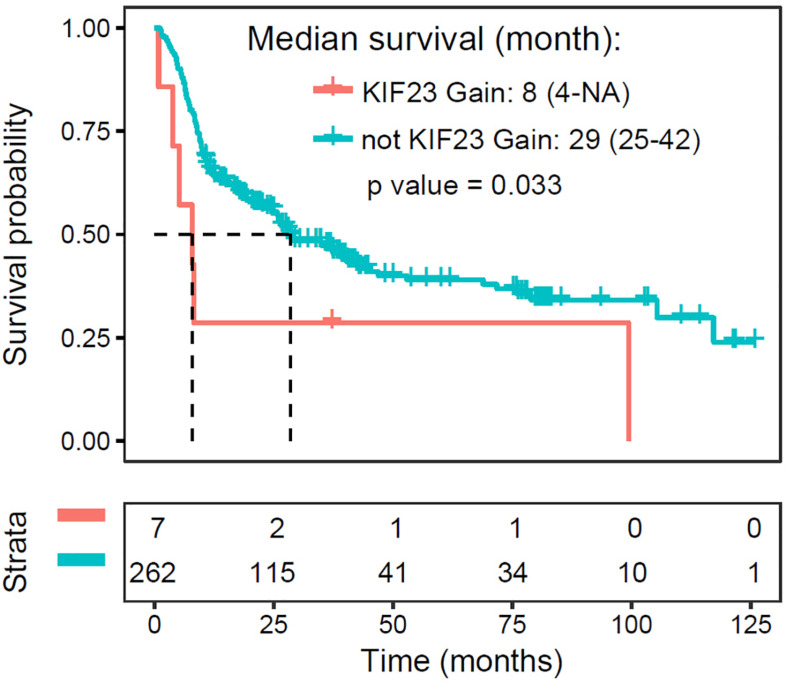
Amplification of KIF23 is a negative prognosticator for glioma patients.

Since higher expression of KIF23 was negatively associated with overall survival with patients, we further investigated the prognostic value of KIF23 amplification with 319 glioma samples. As what was expected (Figure 4), patient with copy number gaining of KIF23 showed significantly worse survival than those without KIF23 amplification (log-rank test, *p* value = 0.033). This result was in consistence with the prognostic value of KIF23 high expression, which consolidates the role of KIF23 in glioma pathophysiology processes.

### Higher Copy Number of KIF23 Is Significantly Associated With TNF-α Signaling Pathway and Cellular Mitotic Activities

**FIGURE 5:**
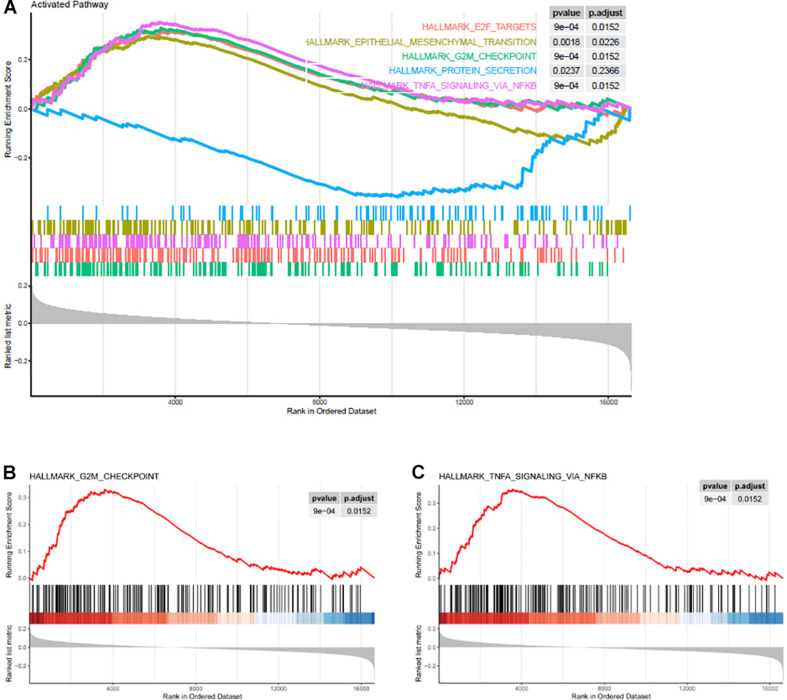
Higher copy number of KIF23 was associated with active cell biological process **(A)**, including TNF-α signaling pathway **(B)**, and mitotic cell circle checkpoint **(C)**.

To explore the underlying mechanism of amplification of KIF23, we conducted gene ontology analysis for KIF23 copy number variation. Enrichment analysis revealed that KIF23 copy number positively associated genes tended to be associated with active cell biological process (Figure 5A), including TNF-α signaling pathway (Figure 5B), and G2M checkpoint (Figure 5C). These results indicated that higher copy number of KIF23 was involved with active immune activities and mitotic cellular activities, which further suggested more malignant biological processes. This is consistent with what we have found in the analysis of expression of KIF23, consolidating the malignant role of KIF23 in glioma.

## Discussion

KIF23, also known as MKLP1 (mitotickinesin-like protein-1), is a member of kinesin-like motor protein superfamily. The protein encoded by KIF23 contains the kinesin superfamily motor domain at the N-terminal region. The domain mainly localizes in the interzone of mitotic spindles and acts as a plus-end-directed motor enzyme that moves along anti-parallel microtubules (Nislow et al., 1992). KIF23 has been identified as a key regulator of cytokinesis for its essential role in spindle midbody formation (Zhu et al., 2005; Liu and Erikson, 2007). Antagonism of KIF23 expression causes cell growth inhibition, and the formation of enlarged cell bodies with binuclear/multinuclear in many tumor cells (Liu et al., 2004; Takahashi et al., 2011; Kato et al., 2016b), probably due to the cell cycle arrest which further caused mitosis failure.

It seems that elevated of KIF23 expression is a common event in various human cancers. KIF23 was reported to be overexpressed in pancreatic ductal adenocarcinoma (Gao et al., 2020), malignant pleural mesothelioma (Kato et al., 2016a), lung cancer (Kato et al., 2016b; Ye et al., 2017), breast cancer (Zou et al., 2014), hepatocellular carcinoma (HCC) (Sun et al., 2015; Cheng et al., 2020), gastric cancer (Li X. L. et al., 2019; Liang et al., 2020), and ovarian cancer (Li T. et al., 2019; Hu et al., 2020). Interestingly, most studies also showed that patients with higher KIF23 expression had worse prognosis survival compared to these with lower KIF23 expression (Kato et al., 2016b; Ye et al., 2017; Li T. et al., 2019; Li X. L. et al., 2019). All these studies indicated that KIF23 plays as an oncogene in cancers. In glioma, KIF23 was also showed to be up-regulated compared to normal brain samples, and inhibition of KIF23 suppressed the proliferation of glioma cells both *in vivo* and *in vitro*. Furthermore, high KIF23 expression also conferred poor survival in glioma patients (Takahashi et al., 2011; Zhao et al., 2018). However, these two studies only employed 11 and 54 glioma samples, respectively. To further validate the above results, we investigated KIF23 expression pattern and its relationship with clinical features in glioma based on 305 samples from CGGA whole genome mRNA expression microarray data in our previous study. The analysis showed that: (1) KIF23 expression was positively correlated with tumor malignancy (grade, wild-type IDH1, G3, and Mesenchymal subtype preference), (2) patients with higher expression of KIF23 had a shorter survival time than those with lower KIF23 expression, and (3) KIF23 was an independent prognostic biomarker for glioma patients. We also demonstrated that reduction of KIF23 expression significantly suppressed U87MG cells proliferation *in vitro* and intracranial tumor growth *in vivo*, as well as prolonged intracranial glioma mice’s overall survival days (Sun et al., 2016). All these researches indicated the potential value of KIF23 as therapeutic target in tumors. Thus, it is urgent and valuable to clarify the signaling pathway of KIF23, as well as the reason which caused its abnormal expression.

One study assumed that mutation in the CHR of KIF23 promoter may cause increased KIF23 expression (Fischer et al., 2013). In another study, Vikberg et al. (2017) employed a mutation screening of the KIF23 in 15 non-small-cell lung cancer (NSCLC) cases with elevated expression level of KIF23, however, none of the examined samples had the mutation in the CHR of KIF23 by using sanger sequencing and single nucleotide polymorphism (SNP)-array. Interestingly, by assessment of CNAs in these samples, they concluded the elevated level of KIF23 might be due to additional copy of chromosome 15. In other researches, KIF23 p.R671W mutation was detected in one family with colorectal cancer (DeRycke et al., 2013). Melanoma cells derived from metastatic lesions patients also found KIF23 mutation by whole-exome sequencing and SNP array profiling (Cifola et al., 2013). Furthermore, KIF23 mutation was detected in about half of 38 tested tumor types (20 out of 38 were confirmed with elevated KIF23 expression). Additionally, CNAs analysis showed that gain in three out of ten tumors, loss in two type of cancer and five tumors confirmed with both gain and loss (Cerami et al., 2012; Vikberg et al., 2017). Based on the hypothesis that the presence of activating somatic mutation or CNAs may cause KIF23 overexpression, we first evaluated the KIF23 expression pattern and prognosis value in CGGA 325 and 693 RNA sequencing database. The results showed that KIF23 expression was positively correlated with tumor grade. Moreover, higher KIF23 expression conferred poor survival, which was consistent with our previous study (Sun et al., 2016). In order to investigate KIF23 somatic mutation and CNAs in glioma, we then screened the 319 glioma samples by using whole-exome sequencing analysis from CGGA database. However, only two case-specific non-synonymous mutations were detected. KIF23 mutation rate was less than 1% in our dataset. Next, we classified the 319 gliomas into CNAs Deletion group, Wildtype group, and Amplification group according to the log ratio. Then, the KIF23 gene expression was calculated, and a positive correlation was detected between KIF23 FPKM expression values and CNA. CNAs associated gene ontology revealed that KIF23 amplification was involved with active immune response and mitotic cell activities, which was consistent with the function of KIF23 expression in our previous paper. These results indicated that DNA copy number amplification may potentially contribute to elevated KIF23 expression in glioma while the biological effects of nucleotide mutation in KIF23 warrants additional investigation.

## Data Availability Statement

The original contributions presented in the study are included in the article/supplementary material, further inquiries can be directed to the corresponding author.

## Author Contributions

L-HS, ZZ, and ZW contributed to study concept and design. ZZ, ZW, Z-SB, W-ZG, and Y-DZ contributed to acquisition of data. C-JR, TL, and YW contributed to analysis and interpretation of data. L-HS, ZZ, and ZW contributed to draft of the manuscript. All authors contributed to the article and approved the submitted version.

## Conflict of Interest

The authors declare that the research was conducted in the absence of any commercial or financial relationships that could be construed as a potential conflict of interest.

## Footnotes

1 http://www.cgga.org.cn

2 http://broadinstitute.github.io/picard/

3 http://cgga.org.cn

4 http://www.gsea-msigdb.org/gsea/index.jsp
